# Predicting Outcome on Admission and Post-Admission for Acetaminophen-Induced Acute Liver Failure Using Classification and Regression Tree Models

**DOI:** 10.1371/journal.pone.0122929

**Published:** 2015-04-17

**Authors:** Jaime Lynn Speiser, William M. Lee, Constantine J. Karvellas, for the US Acute Liver Failure Study Group

**Affiliations:** 1 Department of Public Health Sciences, Medical University of South Carolina, Charleston, South Carolina, United States of America; 2 Division of Digestive and Liver Diseases, Department of Internal Medicine, University of Texas Southwestern Medical Center, Dallas, Texas, United States of America; 3 Divisions of Hepatology and Critical Care Medicine, University of Alberta, Edmonton, Canada; UFMG, BRAZIL

## Abstract

**Background/Aim:**

Assessing prognosis for acetaminophen-induced acute liver failure (APAP-ALF) patients often presents significant challenges. King’s College (KCC) has been validated on hospital admission, but little has been published on later phases of illness. We aimed to improve determinations of prognosis both at the time of and following admission for APAP-ALF using Classification and Regression Tree (CART) models.

**Methods:**

CART models were applied to US ALFSG registry data to predict 21-day death or liver transplant early (on admission) and post-admission (days 3-7) for 803 APAP-ALF patients enrolled 01/1998–09/2013. Accuracy in prediction of outcome (AC), sensitivity (SN), specificity (SP), and area under receiver-operating curve (AUROC) were compared between 3 models: KCC (INR, creatinine, coma grade, pH), CART analysis using only KCC variables (KCC-CART) and a CART model using new variables (NEW-CART).

**Results:**

Traditional KCC yielded 69% AC, 90% SP, 27% SN, and 0.58 AUROC on admission, with similar performance post-admission. KCC-CART at admission offered predictive 66% AC, 65% SP, 67% SN, and 0.74 AUROC. Post-admission, KCC-CART had predictive 82% AC, 86% SP, 46% SN and 0.81 AUROC. NEW-CART models using MELD (Model for end stage liver disease), lactate and mechanical ventilation on admission yielded predictive 72% AC, 71% SP, 77% SN and AUROC 0.79. For later stages, NEW-CART (MELD, lactate, coma grade) offered predictive AC 86%, SP 91%, SN 46%, AUROC 0.73.

**Conclusion:**

CARTs offer simple prognostic models for APAP-ALF patients, which have higher AUROC and SN than KCC, with similar AC and negligibly worse SP. Admission and post-admission predictions were developed.

**Key Points:**

• Prognostication in acetaminophen-induced acute liver failure (APAP-ALF) is challenging beyond admission

• Little has been published regarding the use of King’s College Criteria (KCC) beyond admission and KCC has shown limited sensitivity in subsequent studies

• Classification and Regression Tree (CART) methodology allows the development of predictive models using binary splits and offers an intuitive method for predicting outcome, using processes familiar to clinicians

• Data from the ALFSG registry suggested that CART prognosis models for the APAP population offer improved sensitivity and model performance over traditional regression-based KCC, while maintaining similar accuracy and negligibly worse specificity

• KCC-CART models offered modest improvement over traditional KCC, with NEW-CART models performing better than KCC-CART particularly at late time points

## Introduction

Acetaminophen (APAP) is the most common cause of acute liver failure (ALF) in Europe and North America [1, 2]. Injury and recovery follow a hyper-acute pattern, in which maximum hepatocyte destruction is complete by 72 hours following a one-time ingestion, with potential recovery equally swift. Despite reasonable post-transplant outcomes, liver transplantation (LT) for acetaminophen-induced acute liver failure (APAP-ALF) often presents significant challenges in management due to the rapidity and severity of illness, the potential for recovery without LT and the presence of complex psychosocial issues in most patients [3, 4]. Data from the US ALFSG shows that approximately 25% of APAP patients are listed for LT and less than 10% receive LT [5]. Current data suggest that APAP recovery for many patients is determined by 3–4 days following onset of illness [6]. However, with advances in intensive care unit (ICU) management, such as continuous renal replacement therapy (RRT) and neuroprotective strategies, many patients who would otherwise have succumbed may remain alive for longer periods with intensive care well beyond the initial insult [7, 8]. While the King’s College Criteria (KCC) [9] and Clichy criteria [10] have been validated on admission, prediction of outcome at later time points appears less accurate [11] when hepatic dysfunction would be characterized primarily by immunosuppression rather than multi-organ failure [8].

Numerous subsequent studies that have utilized KCC have shown, for the most part, relatively poor sensitivity of the APAP criteria, ranging between 25% and 76%—many patients who did not meet criteria still died during the incident hospitalization [2, 12–14]. Similarly, a specificity of 80–90% implies that some patients may have a good outcome despite meeting KCC and potentially could undergo unnecessary LT [13, 15]. None of these studies has evaluated KCC using daily (serial) time points.

The primary aim of this study was to explore the use of Classification and Regression Tree (CART) methodology to determine prognosis for use early (at admission) or post-admission (days 3–7) in APAP-ALF patients. The CART methodology [16, 17] allows the development of predictive models using binary splits on variables which can be read like a flow chart. Gaining popularity in diverse medical fields [18, 19], CART models offer an intuitive method for predicting outcome, using processes familiar to clinicians (e.g. “high” versus “low” values of a predictor). We hypothesized that CART models would have similar or modestly higher predictive accuracy, sensitivity, specificity, and area under the receiver-operating curve (AUROC) compared to traditional KCC. Specifically, the objectives for this study were:

1. To develop CART models containing the same variables as traditional KCC (pH, INR, creatinine, coma grade) to predict death/LT at 21 days for APAP-ALF patients using training datasets on admission and post-admission (days 3–7).
2. To develop CART models containing variables suggested within current literature to predict death/LT at 21 days for APAP-ALF patients using training datasets on admission and post-admission (days 3–7).
3. To compare the predictive accuracy, sensitivity, specificity, and AUROC for traditional KCC, CART models using KCC variables (KCC-CART), and CART models using literature-suggested variables (NEW-CART).

## Materials and Methods

### Study Design

Data from 803 APAP-ALF patients enrolled within the ALF Study Group (ALFSG) database from January 1998 to September 2013 (25 sites overall, 14 currently active; see acknowledgments) were used in this retrospective cohort study. The authors’ Institutional Review Board (IRB)/Health research ethics boards of all enrolling US ALFSG sites have approved all research and all clinical investigation has been conducted according to the principles expressed in the Declaration of Helsinki. Consent/assent were obtained from all patients/their next of kin for collection of data in the US ALFSG registry. Patient records/information was anonymized and de-identified prior to use in this analysis. Participants who were medically competent provided written informed consent to participate in this study. In cases when patients were unable to provide written consent (critical illness, hepatic encephalopathy) written assent was obtained by the next of kin. Upon regaining capacity, patients were given the option to withdraw written consent. In those cases, data were not included in the registry. Documentation of participant consent/assent is kept in duplicate at individual sites of the US ALFSG (e.g. University of Alberta). Health research ethics boards/ Institutional review boards at all sites of the US ALFSG have approved this consent procedure.

### Participants

ALFSG registry eligibility criteria include: a) hepatic encephalopathy of any degree; b) evidence of moderately severe coagulopathy (international normalized ratio (INR) greater than or equal to 1.5); c) presumed acute illness onset of less than 26 weeks; and d) no cirrhosis [20]. For this study, only patients within the ALFSG registry with primary diagnoses of APAP determined by the site investigator were eligible. Eligible patients with missing or unknown 21-day outcome data were excluded in the final analysis.

### Operational Definitions

**Hepatic encephalopathy (HE) grade** was defined using the West Haven Criteria (summarized); grade 1: any alteration in mentation, grade 2: being somnolent or obtunded but easily rousable or presence of asterixis, grade 3: being rousable with difficulty and, grade 4: unresponsive to deep pain [21]. In this study we defined ‘low coma grade’ as grade 1 or 2 and ‘high coma grade’ as grade 3 or 4. The **KCC** [9] predicts poor outcome (death/transplant) if: a) pH is less than 7.3 or b) if INR is greater than 6.5, creatinine is greater than 3.4 mg/dL, and coma grade is high. The **model for end stage liver disease** (MELD) is defined as 10*(0.957*log(4)+0.378*log(bilirubin)+1.12*log(INR)) for dialyzed patients and 10*(0.957*log(creatinine)+0.378*log(bilirubin)+1.12*log(INR)) for patients not dialyzed [22]. For evaluating the predictive performance of the models, **specificity** (SP) is the proportion of correctly predicted poor outcomes (death/transplant) and **sensitivity** (SN) is the proportion of correctly predicted good outcomes (spontaneous survival).

### CART Analysis

CART analysis is a machine-learning algorithm, in which a model is developed using deterministic rules. The nonparametric nature of CART offers results which are simple to use (e.g. does not require calculation or use of an application) and interpret (e.g. low versus high variable values) and a framework with few assumptions. These aspects of CART are advantageous compared to logistic regression, where calculations may be cumbersome (e.g. plugging in numbers and exponentiation requires a calculator or application), interpretation of results may be unclear (e.g. if there are interactions between two or more predictors), and assumptions may not be satisfied. Detailed information on how trees are developed is included in the Supplementary Methods (S2 File). Trees are read from top to bottom like a flow chart in order to gain a prediction for 21-day status. Starting at the top of the tree, one follows the branch corresponding to observed variable values of a patient until a terminal node has been reached, in which the fraction of patients contained in each outcome category is displayed. These tables may be used to assess the likelihood that a patient will fall within each outcome group.

### Variables

The main outcome of interest was death/LT 21 days after ALFSG enrollment. Variables used for traditional KCC and KCC-CART prediction at admission included pH, INR, creatinine, and hepatic coma grade (high versus low). For the post-admission traditional KCC and KCC-CART prediction, pH was excluded since many patients received RRT in the days following admission to the hospital. Potential variables considered for the admission NEW-CART models included age, sex, coma grade (high versus low), platelets, INR, bilirubin, pH, ammonia (venous or arterial), creatinine, lactate, phosphate, aspartate transaminase (AST), alanine transaminase (ALT), MELD, mechanical ventilation (MV), vasopressor use, and RRT. These same variables were also considered for the post-admission NEW-CART models, excluding pH.

### Statistical Methods

Admission CART models were constructed using a training dataset (N = 288) and were assessed using a test dataset (N = 515). Post-admission CART models were created using data from days 3–7 within the ALFSG registry. The dataset was constructed using a population averaged approach [23], which included the average of continuous variables over days 3–7 and composite categorical variables for each patient. Training data (N = 146) and test data (N = 354) were randomly split. We aimed to develop CART models that offered higher sensitivity, while maintaining similar overall accuracy, compared to traditional KCC by using a weighted sampling scheme to split admission and post-admission datasets. After trees were created, nodes containing few observations were removed, a process known as pruning, by selecting the complexity parameter, which minimized the cross-validated error rate. Details of CART construction are described in the Supplementary Methods (S2 File).

Prediction of death/LT using KCC was determined at admission for 679/803 (85%) APAP-ALF patients with complete data for predictor variables (pH, INR, creatinine, and coma grade) and outcome data. Post-admission KCC was assessed for 341/803 (43%) APAP-ALF patients with complete day 3 data. The calculation of KCC required non-missing data for all three variables (INR, creatinine, and coma grade), and missing values increased with time because in-patient data collection halted with patients’ death, hospital discharge or receipt of LT. Thus, only day 3 data were included for the traditional KCC model to maximize sample size. KCC was calculated on admission and post-admission for the entire dataset and for the training and testing datasets used to create the CART models for patients with non-missing KCC variables.

Analyses were completed using SAS Version 9.3 (SAS Institute, Cary, NC) and R software [24]. The R package rpart was used to create the CART models [25]. Traditional KCC, KCC-CART and NEW-CART models were assessed in terms of overall accuracy, sensitivity and specificity using binomial estimates and confidence intervals in R [24]. AUROC for the CART models was determined using the R package ROCR [26].

## Results

### Patient Characteristics

**Fig 1:**
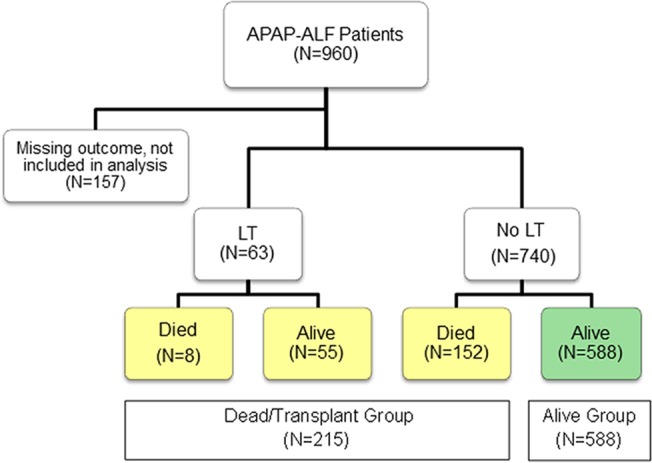
ALFSG Subjects in this Study. There were 588 APAP-ALF subjects who spontaneously survived, and 215 subjects who had a LT or died by day 21.

Demographic and clinical characteristics of patients are displayed in Table 1 for the admission training and testing datasets. Of the 803 APAP-ALF patients (within both training and testing datasets), the median age was 37 (29–47) years and 76% were female. A total of 157 patients had missing outcome data. Fig 1 illustrates a flow chart of ALF-APAP patients included in this study. One hundred and eighty-eight (23%) patients were listed for transplant and 63 (8%) received a LT. Overall, 588 (73%) patients recovered and 152 (19%) died without a LT, while 55 (87%) of the LT patients were alive at the end of follow-up and had been transplanted by 21 days. Thus, the dead/LT outcome group contained 215 (27%) of all ALF-APAP patients. The median time from ALFSG enrollment to death was 4 days (Interquartile Range (IQR): 2–16) and the median time from ALFSG enrollment to transplant was 2 days (IQR: 1–3).

**Table 1:** Demographic and Clinical Characteristics for 803 APAP ALF Patients.

	Admission Training Dataset Subjects (N = 288)		Admission Testing Dataset Subjects (N = 515)		All ALF-APAP Subjects (N = 803)	
	N	Number (%) or Median (IQR)	N	Number (%) or Median (IQR)	N	Number (%) or Median (IQR)
**Death/LT by day 21**	288	144 (50%)	515	71 (13.8%)	803	215 (26.8%)
**Age**	288	39.0 (30.0–47.0)	515	36.0 (28.0–47.0)	803	37.0 (29.0–47.0)
**Sex (female)**	288	222 (77.1%)	515	389 (75.5%)	803	611 (76.1%)
**Race**	288		515		803	
White		246 (85.4%)		451 (87.6%)		697 (86.8%)
African-American		30 (10.4%)		36 (7.0%)		66 (8.2%)
Other		12 (4.2%)		35 (6.8%)		47 (5.0%)
**Admission day NAC (oral or IV)**	288	255 (88.5%)	515	456 (88.5%)	803	711 (88.5%)
**Admission Coma Grade (I or II)**	288	116 (40.2%)	515	232 (45.0%)	803	348 (43.3%)
**Admission biochemistry**						
Hemoglobin (g/dl)	285	10.4 (9.4–12.3)	512	11.2 (9.5–12.6)	797	10.8 (9.5–12.5)
White Blood count (x10^9^/L)	284	9.3 (6.6–13.9)	513	9.3 (6.4–14.1)	797	9.3 (6.4–14.0)
Platelet count (x10^9^/L)	284	123.5 (76.0–183.5)	510	128.0 (84.0–181.0)	794	126.0 (82.0–182.0)
INR	282	3.1 (2.0–4.6)	506	2.8 (2.0–4.3)	788	2.9 (2.0–4.4)
AST (IU/L)	288	4570.5 (1520.0–8762.5)	508	3473.0 (1302.0–7352.0)	796	3686.5 (1350.5–7980.0)
ALT (IU/L)	287	3520.0 (1847.0–5778.0)	507	3743.0 (1967.5–6449.5)	794	3685.5 (1959.0–6218.0)
Bilirubin (mg/dl)	285	4.4 (2.7–6.7)	508	4.4 (2.8–6.4)	793	4.4 (2.8–6.4)
pH	257	7.4 (7.3–7.5)	440	7.4 (7.4–7.5)	697	7.4 (7.3–7.5)
Ammonia (venous; μmol/L)	96	124.5 (82.5–176.5)	185	91.0 (59.5–142.5)	281	102.0 (68.0–153.0)
Creatinine (mg/dL)	287	2.0 (1.0–3.5)	513	1.7 (0.9–3.2)	800	1.9 (0.9–3.4)
Lactate (mmol/L)	182	5.7 (3.3–11.4)	309	3.6 (2.1–7.3)	491	4.5 (2.4–8.7)
Phosphate (mg/dL)	254	2.9 (1.9–4.2)	459	2.4 (1.6–3.5)	713	2.6 (1.7–3.7)
PO2/FiO2 ratio	219	3.6 (2.4–4.7)	379	3.8 (2.7–4.7)	598	3.7 (2.5–4.7)
MELD (admission)	280	26.9 (18.4–34.1)	500	24.9 (15.7–31.1)	780	25.7 (16.7–32.3)
**Organ support (7-days)**	288		515			
Mechanical ventilation		206 (71.5%)		330 (64.1%)	803	536 (66.7%)
Vasopressors		127 (44.1%)		138 (26.8%)	803	265 (33.0%)
Renal Replacement therapy		86 (29.9%)		158 (30.7%)	803	244 (30.4%)
**ICP therapies (7-days)**	288		515			
Mannitol		84 (29.2%)		101 (19.6%)	803	185 (23.0%)
Hypertonic saline		28 (9.7%)		39 (7.6%)	803	67 (8.3%)
Barbiturates		25 (8.7%)		41 (8.0%)	803	66 (8.2%)
Hypothermia		23 (8.0%)		34 (6.6%)	803	57 (7.1%)
Sedatives		207 (71.9%)		343 (66.6%)	803	550 (68.5%)
**Blood products (7-days)**	288		515			
Fresh Frozen Plasma		171 (59.4%)		255 (49.5%)	803	426 (53.1%)
Recombinant VIIA		8 (2.8%)		8 (1.6%)	803	16 (2.0%)
Platelets		79 (27.4%)		88 (17.1%)	803	169 (21.0%)

***Abbreviations***

N: frequency; IQR: inter-quartile range; MELD: Model for End-Stage Liver Disease.

Coma grade as defined by West Haven Criteria [21]: Low grade ~ Grade I or II, High grade ~ Grade III or IV

### Admission Models

**Fig 2:**
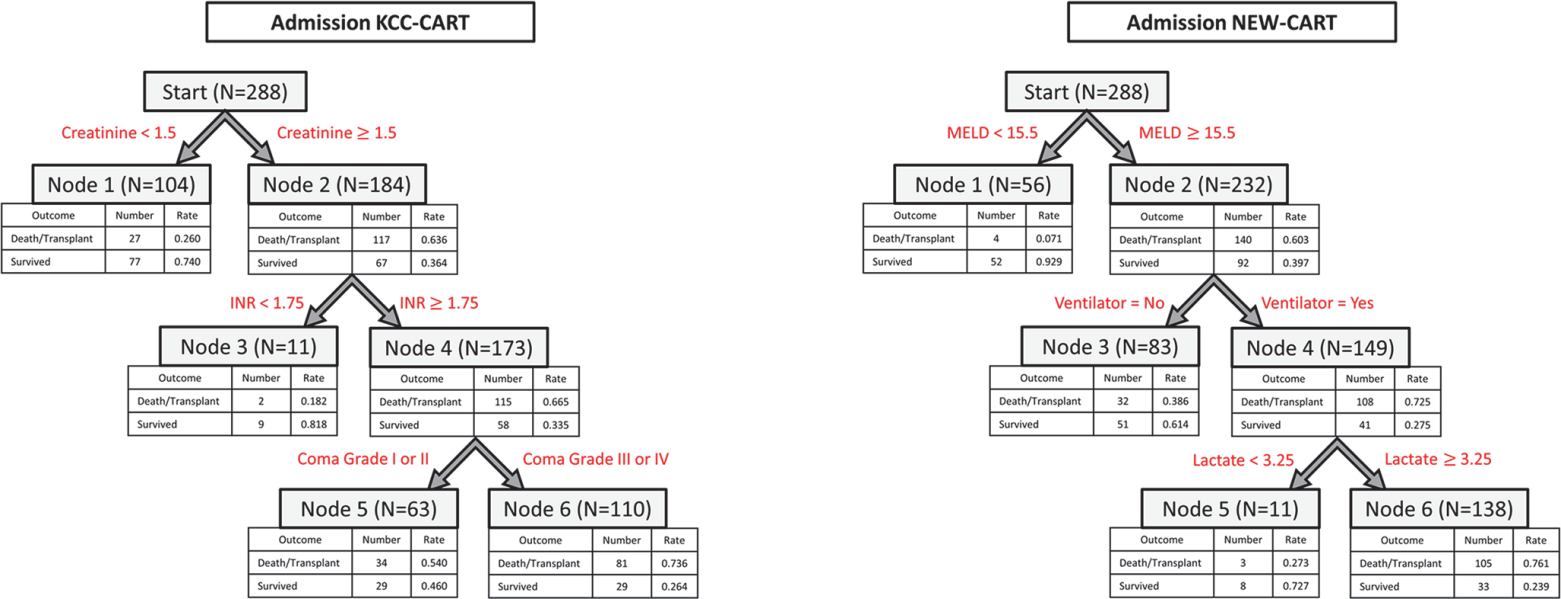
Admission CART Models. The admission KCC-CART (left panel) has three decision rules and consists of six total nodes. Each node provides the total number of subjects within the node, as well as the number of survivors and dead/LT patients with the respective rates. Node 6 represents high risk of dead/LT outcome, nodes 1 and 3 are low risk of dead/LT outcome, and node 5 is moderate risk of dead/LT outcome. To calculate performance measures for the model, all subjects in nodes 5 and 6 were predicted as dead/LT outcomes, and all subjects in nodes 1 and 3 were predicted as spontaneous survivors. The admission NEW-CART (right panel) also has three decision rules and consists of six total nodes. Node 6 patients were considered high risk for dead/LT outcome and were predicted as such, whereas nodes 1, 3 and 5 were predicted as survivors.

The KCC-CART model for admission is depicted in the left panel of Fig 2. Variables included within the KCC-CART admission model included creatinine, INR and coma grade (high versus low) for a total of three splitting points. The NEW-CART model for admission, which considered as possible predictors the eighteen variables listed previously, is shown in the right panel of Fig 2. Selected variables for the NEW-CART admission model were MELD, MV, and lactate, with one splitting point for each of these.

Performance measures and their associated confidence intervals for the three admission prognosis models are presented in Table 2. Traditional KCC had good overall predictive accuracy of 69% and specificity of 90%, while sensitivity was low (27%). AUROC of 0.58 indicated poor model predictive ability. For the admission training dataset, traditional KCC offered poor accuracy (58%), sensitivity (53%) and AUROC (0.56), with moderate specificity (76%). Accuracy and specificity were significantly higher for KCC using the testing dataset (80% and 87% respectively); however, the sensitivity remained quite low (29%). The admission KCC-CART offered lower predictive accuracy (66%) and specificity (65%) for the testing dataset; however, the predicted sensitivity (67%) and AUROC (0.70) were increased. The predictive accuracy of KCC using all admission data and KCC-CART were not statistically different (indicated since confidence intervals overlap), but the AUROC and sensitivity were significantly higher for the KCC-CART (confidence intervals do not overlap). KCC demonstrated significantly higher specificity than KCC-CART for the test dataset. Marginal improvement was made over KCC-CART by NEW-CART model, with predictive accuracy of 72%, specificity of 71%, sensitivity of 77% and AUROC 0.76, though differences were not significant, indicated by overlapping confidence intervals. Overall, CART models performed statistically better than traditional KCC using all admission data in terms of sensitivity and AUROC while maintaining similar predictive accuracy and significantly lower specificity.

**Table 2:** Admission Day Model Performance for patients with Acetaminophen-induced Acute Liver Failure.

Admission Model	Variables in Model	Dataset Used (Sample size)	Accuracy (95% CI)	Specificity (95% CI)	Sensitivity (95% CI)	AUROC (95% CI)
KCC	pH, INR, creatinine, coma grade (low/high)	All Day 1 with non-missing values for all variables (N = 679)	0.692 (0.656–0.727)	0.895 (0.864–0.922)	0.272 (0.214–0.335)	0.582 (0.551–0.613)
KCC	pH, INR, creatinine, coma grade (low/high)	Day 1 Training Set (N = 249)	0.578 (0.514–0.640)	0.759 (0.624–0.865)	0.528 (0.456–0.600)	0.559 (0.528–0.590)
KCC	pH, INR, creatinine, coma grade (low/high)	Day 1 Test Set (N = 424)	0.804 (0.763–0.841)	0.870 (0.831–0.902)	0.292 (0.170–0.441)	0.585 (0.554–0.616)
KCC-CART	INR, creatinine, coma grade (low/high), pH	Day 1 Training Set (N = 288)	0.722 (0.667–0.773)	0.715 (0.634–0.787)	0.729 (0.649–0.800)	0.740 (0.712–0.767)
KCC-CART	INR, creatinine, coma grade (low/high), pH	Day 1 Test Set (N = 515)	0.658 (0.615–0.699)	0.652 (0.605–0.696)	0.670 (0.580–0.801)	0.704 (0.675–0.732)
NEW-CART	MELD, MV, lactate	Day 1 Training Set (N = 288)	0.750 (0.696–0.800)	0.771 (0.693–0.837)	0.729 (0.649–0.800)	0.791 (0.764–0.816)
NEW-CART	MELD, MV, lactate	Day 1 Test Set (N = 515)	0.718 (0.677–0.757)	0.710 (0.666–0.752)	0.767 (0.654–0.858)	0.755 (0.727–0.781)

**Abbreviations**

AUROC: Area under the receiver operator curve

CART: Classification and Regression Tree Analysis

CI: Confidence interval

Coma grade as defined by West Haven Criteria[21]: Low grade ~ Grade I or II, High grade ~ Grade III or IV

KCC: King’s College Criteria

KCC-CART: Classification and Regression Tree analysis using traditional King’s College Criteria Variables

INR: Internationalized Ratio; MELD: Model for End-Stage Liver Disease, MV: mechanical ventilation.

N: Number of patients in sample dataset with outcomes; 95% CI: 95% confidence intervals

NEW-CART: Classification and Regression Tree analysis using new Variables

### Post-Admission Models

**Fig 3:**
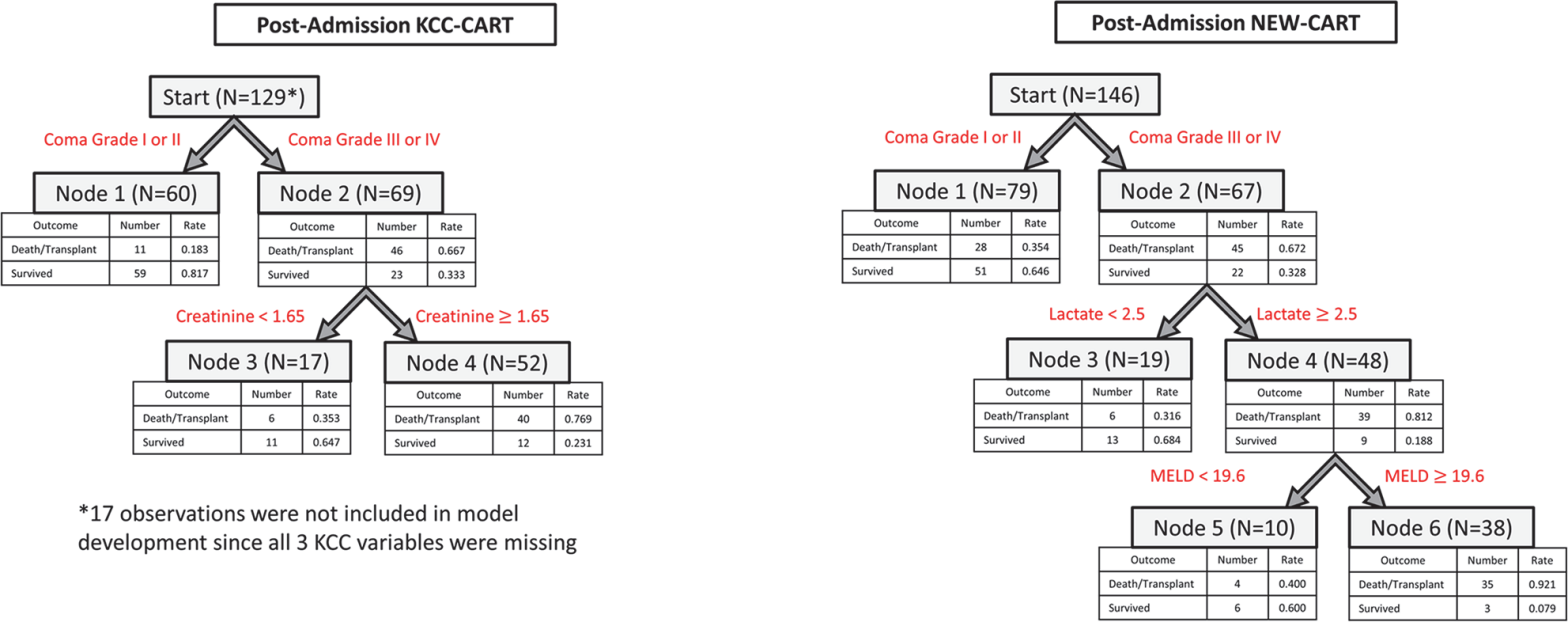
Post-Admission CART Models. The post-admission KCC-CART (left panel) has two decision rules and consists of four total nodes. Each node provides the total number of subjects within the node, as well as the number of survivors and dead/LT patients with the respective rates. Node 4 represents high risk of dead/LT outcome, and nodes 1 and 3 are low risk of dead/LT outcome. To calculate performance measures for the model, all subjects in node 4 were predicted as dead/LT outcomes, and all subjects in nodes 1 and 3 were predicted as spontaneous survivors. The post-admission NEW-CART (right panel) has three decision rules and consists of six total nodes. Node 6 patients were considered high risk for dead/LT outcome and were predicted as such, whereas nodes 1, 3 and 5 were predicted as survivors.

The KCC-CART post-admission model is illustrated in the left panel of Fig 3. Variables included within the KCC-CART day 3–7 model were creatinine and coma grade (high versus low), with only two splitting points. The NEW-CART model for post-admission, which considered as possible predictors the seventeen variables listed previously, is presented in the right panel of Fig 3. Variables selected for NEW-CART post-admission were coma grade (high versus low), lactate, and MELD, with one splitting point for each of these.

**Fig 4:**
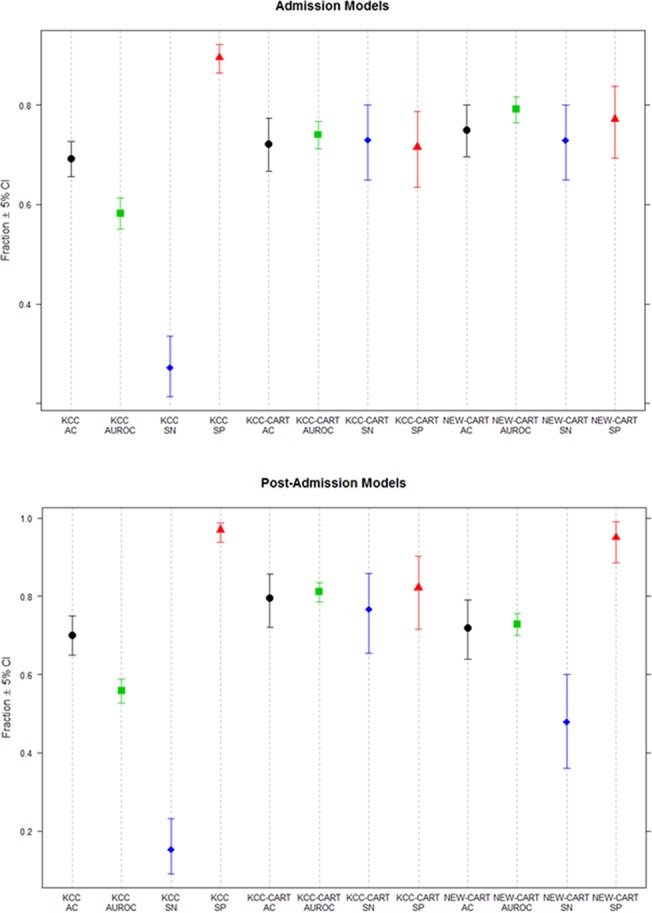
Plots of Confidence Intervals of Accuracy, AUROC, Sensitivity and Specificity for Admission and Post-Admission Models. Plots display confidence intervals for accuracy (AC), area under the receiver operating curve (AUROC), sensitivity (SN) and specificity (SP) from Table 2. The admission plot (top panel) illustrates non-overlapping, higher confidence intervals for both KCC-CART and NEW-CART compared to KCC for AUROC and sensitivity, and lower confidence intervals for specificity. This indicates that the CART models had significantly better AUROC and sensitivity than KCC, but had significantly worse specificity compared to KCC. Confidence intervals for accuracy for KCC, KCC-CART and NEW-CART all overlap, indicating no significant differences between the models. The post-admission plot (bottom panel) again indicated no significant differences between the models in terms of accuracy; however, the AUROC and sensitivity of KCC-CART was significantly higher than that of NEW-CART and KCC. The specificity of KCC was highest, but did no differ significantly from that of the NEW-CART. KCC demonstrated significantly higher specificity than the KCC-CART, but the difference was not significant compared to the NEW-CART.

Table 3 displays performance measures and their associated confidence intervals for the three post-admission prognosis models (see also Fig 4). The traditional KCC had low sensitivity (15%) and AUROC (0.56), but maintained an overall prediction accuracy of 70% and specificity of 97% using all post-admission data. Accuracy of KCC for the post-admission testing dataset was significantly higher (86%), with similar values for specificity, sensitivity and AUROC. The KCC-CART developed for post-admission had similar prediction accuracy (82%), slightly lower specificity (86%) and higher sensitivity (46%), with AUROC of 0.72 indicating improvement in prediction compared to traditional KCC for the test dataset. Accuracy for KCC and KCC-CART were comparable (confidence intervals overlap), but KCC-CART had significantly higher AUROC and sensitivity than KCC, and KCC had significantly higher specificity than KCC-CART (non-overlapping confidence intervals). For post-admission prediction, the NEW-CART model offered marginal improvement over traditional KCC, with prediction accuracy of 86%, specificity of 91% and sensitivity of 46%. AUROC for NEW-CART was 0.68, which was lower than that of the KCC-CART for post-admission prediction. Overlapping confidence intervals for accuracy and specificity indicate no significant differences between KCC-CART and NEW-CART, but non-overlapping confidence intervals for KCC-CART demonstrated significantly higher AUROC and sensitivity compared to NEW-CART.

**Table 3:** Post-Admission Model Performance for patients with Acetaminophen-induced Acute Liver Failure.

Post-Admission Model	Variables in Model	Dataset Used (Sample size)	Accuracy (95% CI)	Specificity (95% CI)	Sensitivity (95% CI)	AUROC (95% CI)
KCC	pH, INR, creatinine, coma grade (low/high)	All Day 3 with non-missing values for all variables (N = 341)	0.701 (0.649–0.749)	0.969 (0.938–0.988)	0.152 (0.091–0.232)	0.558 (0.527–0.589)
KCC	pH, INR, creatinine, coma grade (low/high)	Day 3–7 Training Set (N = 99)	0.556 (0.452–0.655)	1.000 (0.933–1.000)	0.043 (0.005–0.148)	0.522 (0.491–0.553)
KCC	pH, INR, creatinine, coma grade (low/high)	Day 3–7 Test Set (N = 212)	0.863 (0.809–0.906)	0.984 (0.953–0.997)	0.103 (0.022–0.274)	0.544 (0.513–0.575)
KCC-CART	creatinine, coma grade (low/high)	Day 3–7 Training Set (N = 146)	0.795 (0.720–0.857)	0.822 (0.715–0.902)	0.767 (0.654–0.858)	0.811 (0.785–0.835)
KCC-CART	creatinine, coma grade (low/high)	Day 3–7 Test Set (N = 354)	0.822 (0.778–0.860)	0.864 (0.822–0.900)	0.459 (0.295–0.631)	0.720 (0.691–0.748)
NEW-CART	coma grade (low/high), lactate, MELD	Day 3–7 Training Set (N = 146)	0.719 (0.639–0.790)	0.959 (0.885–0.991)	0.479 (0.361–0.600)	0.729 (0.700–0.756)
NEW-CART	coma grade (low/high), lactate, MELD	Day 3–7 Test Set (N = 354)	0.859 (0.818–0.893)	0.905 (0.868–0.935)	0.459 (0.295–0.631)	0.680 (0.650–0.709)

**Abbreviations**

AUROC: Area under the receiver operator curve

CART: Classification and Regression Tree Analysis

CI: Confidence interval

Coma grade as defined by West Haven Criteria[21]: Low grade ~ Grade I or II, High grade ~ Grade III or IV

KCC: King’s College Criteria

KCC-CART: Classification and Regression Tree analysis using traditional King’s College Criteria Variables

INR: Internationalized Ratio; MELD: Model for End-Stage Liver Disease, MV: mechanical ventilation.

N: Number of patients in sample dataset with outcomes; 95% CI: 95% confidence intervals

NEW-CART: Classification and Regression Tree analysis using new Variables

### Comparison of Admission and Post-Admission Models

The most important predictor of outcome on admission for the KCC-CART model was creatinine level, followed by INR, then coma grade. For the post-admission KCC-CART, variables that best predicted outcome were coma grade and creatinine, in that order. INR was not selected by the model for inclusion within the post-admission KCC-CART, suggesting it may not be as influential in determining outcome for post-admission patients compared to admission. The KCC-CART at admission was slightly more complex than the model for post-admission prediction. The splitting point determined by the KCC-CART for creatinine in both models was similar: on admission creatinine was split at 1.5 mg/dL and post-admission was split at 1.7 mg/dL. The splitting point for coma grade, a categorical variable, was identical for both models. This indicated that creatinine and coma grade were stable variables to consider in the prediction of outcome post-admission.

For the NEW-CART models, similar but not identical variables were selected by the model for both admission and post-admission. MELD was most important in predicting outcome on admission, with use of MV and lactate levels also influencing the model. For the post-admission NEW-CART model, the best predictors were coma grade, then lactate and MELD. Splitting points were slightly different for variables within the admission and post-admission models. On admission, MELD was split at 16 and lactate at 3.3 mmol/L, whereas post-admission MELD was split at 20 and lactate at a level of 2.5 mmol/L. Coma grade was more important for the post-admission CART compared to the admission model, when MV was a better predictor of outcome. Comparisons of all models by performance characteristics (sensitivity, specificity, accuracy and c-statistic (AUROC)) are shown in Fig 4.

## Discussion

### Key Results

In this analysis, CART models modestly increased predictive performance of 21-day death/LT compared to traditional regression-derived KCC using United States ALFSG data. KCC-CART trees improved considerably the sensitivity and AUROC and provided similar predictive accuracy compared to traditional KCC, while NEW-CART models provide further but marginal improvement over KCC-CART models on admission. In general, the KCC-CART and NEW-CART models are accurate and simple since they do not require cumbersome calculations to obtain a prediction. Interpretation is straightforward in CART analysis, in which “high” and “low” values of predictors are assessed based on data-driven cut points determined by the method. Trees were developed for both admission and post-admission (Figs 2 and 3), providing clinicians an efficient method for assessing prognosis of patients throughout hospitalization. A specific example of how the CART models may be used in practice is presented within the S2 File.

It was somewhat surprising that, compared to the admission day prognosis models; the post-admission models actually demonstrated much lower sensitivity. This likely occurred because data must be censored following death, LT or hospital discharge. Therefore, the post-admission dataset does not contain patients who had any of these events in the first two days. The outcome of death or transplant becomes rarer within the post-admission dataset, thereby making it more difficult for a model to accurately predict the outcome for this group. Given the statistical challenges of predicting rare outcomes, the increased sensitivity of the post-admission CART models compared to traditional KCC illustrates the flexibility of tree modeling.

CART models provide an alternative to traditionally used prognosis models; however, determining the best model is a complex process. NEW-CART models developed in this study offer similar predictive performance on admission compared to traditional KCC and KCC-CART, but included MELD, which must be calculated prior to prediction. Though NEW-CART models only used three variables, there were an additional three variables used in determining MELD (INR, creatinine, and bilirubin). This made the NEW-CART models more complex compared to KCC-CART, which only contained three variables for the admission model and two for the post-admission model (no INR). However, the extra variables needed to calculate MELD are readily available in practice, only requiring slightly more effort for the improved prediction of outcome. A limitation of the post-admission KCC-CART model was that it used creatinine without considering whether or not patients were on RRT. Therefore, the post-admission NEW-CART model may be preferable for prediction compared to the KCC-CART. In summary, both KCC-CART and NEW-CART models have benefits and could be used in predicting death/LT of new APAP-ALF patients for admission and post-admission.

### Comparisons with Previous Studies

In this study, a mechanism for predicting the likelihood of 21-day death/LT in later stages of hospitalization was developed, which is novel since most prognostic models were constructed using hospital admission data. Previous admission prognosis models by Bernal [14] and Schmidt [13] considered lactate and MELD respectively, which were also selected for inclusion in the NEW-CART models. The cut point for lactate was similar: NEW-CART splits lactate at 3.3 mmol/L and Bernal’s criteria splits lactate at 3.5 mmol/L [14]. For MELD, NEW-CART used a cutoff of 15.5, whereas Schmidt used 33 [13]. Phosphate was included in another prognosis model by Schmidt [12] and ammonia was used by Clemmesen [27], but these were not selected for the CART models. Consistent with a study by Audimoolam [28] which found a significant association between the number of MV days and outcome, NEW-CART identified MV as a predictor of prognosis of ALF-APAP patients at admission.

Although it has many benefits compared to other modeling techniques, CART has rarely been implemented in the ALF setting. Nakayama [29] used CART modeling to predict outcome for non-APAP ALF patients. Models developed in this study for the APAP-ALF subgroup achieved slightly better predictive accuracy on admission, with higher predictive accuracy for the post-admission model compared to models developed by Nakayama [29]. Moreover, the prognosis CART models for APAP-ALF patients in this study included fewer predictor variables.

### Limitations

Though CART models offer an alternative to current prognosis criteria, there are some limitations of this study, which should be considered. First, data used to develop and assess new models were from the North American-derived ALFSG registry, so models may not be appropriate for populations elsewhere. Given the orphan status of ALF, it is difficult to find robust external datasets that have many patients with serially collected clinical features. However, trees were created using statistical techniques—pruning and internal validation—(discussed in the S1 File) to address the issue of generalizability. Therefore, it is hypothesized that the CART models should perform well with other populations of APAP-ALF patients. Also, 157 patients (16% of all APAP-ALF patients within the ALFSG database) had to be excluded from model development since their outcome data was missing or unknown. It is possible that this could have introduced bias into the study, though it is feasible to assume that the patients with missing outcomes were similar to patients with non-missing outcomes since no significant differences in variables were detected (data not shown). A limitation of the outcome variable was being defined as spontaneous survival or death/LT, which made the assumption that if a patient, did not receive a LT then he or she would have died. While this may have introduced bias, the structure of the data collection did not allow for the separation of these two outcomes since after LT in-patient data is not recorded. Grouping death and LT into one outcome category has been implemented in many studies [2, 8, 13]. Comparing CART to traditional KCC model performance was limited by the fact that traditional KCC models were limited by missing data in component variables unlike CART. Some limitations to CART modeling as described in the supplemental materials (S2 File) are: determining parameters for model building, possible variability of CART models, and the lack of repeated measures framework. These are minimal compared to the benefits of CART, namely, simplicity of models and high predictive accuracy. Given these limitations, it would be beneficial to use external datasets to validate the CART models developed in this study. Additionally, future incorporation of biomarkers of hepatic regeneration may improve upon models for prognosticating ALF.

## Conclusions

CART models were produced for determining the likelihood of 21-day death/LT both on admission and post-admission for APAP-ALF patients. Offering a simple and accurate method for assessing death/LT at day 21, KCC-CART and NEW-CART models provided an alternative to traditional KCC. Data from the ALFSG registry suggests that CART prognosis models offer similar accuracy, higher sensitivity and AUROC, and lower specificity compared to KCC, but additional datasets should be used to validate these findings.

## Supporting Information

S1 File STROBE guideline for reporting retrospective studies. This file contains the format used for the current study (BMJ 2007).
(DOC)

S2 File Supplementary Methods. This file contains additional information about the CART procedure, CART models created within this study, example interpretation/use of the CARTs, and limitations of CART models.
(DOCX)
