# Supplementary material for: Predicting Outcome on Admission and Post-Admission for Acetaminophen-Induced Acute Liver Failure Using Classification and Regression Tree Models
Source: PLoS One. 2015 Apr 17;10(4):e0122929. doi: 10.1371/journal.pone.0122929 (PMC4401567; doi:10.1371/journal.pone.0122929)
Supplement: S2 File — This file contains additional information about the CART procedure, CART models created within this study, example interpretation/use of the CARTs, and limitations of CART models. (DOCX) [file pone.0122929.s002.docx]

**S2 File: Supplementary methods**

**Benefits of CART**

Constructing optimal binary splits on independent variables to predict outcome, CART is simple to apply in practice and offers reliable accuracy. Compared to traditional categorical prediction models such as logistic regression and linear discriminant analysis, CART has few statistical assumptions. No requirements about the distribution of variables are necessary within this framework. CART also provides an effective way of handling observations with missing predictors in the data and allows for inclusion of variables with complex interactions. Modern advances in computing make CART modeling efficiently implemented using many standard statistical software platforms.

**Tree Development**

Trees were constructed firstly by selecting the variable that optimally separated outcome groups, and a binary split was made. Then, from both of these subgroups, subsequent variables were selected with replacement (meaning that variables can be used more than once within a model) that optimally separated outcome groups, and second levels of binary splits were made. Variable splits were made recursively until stopping criteria were reached, in which case a terminal node occurred. At each terminal node was the outcome prediction for the specific subset of the data.

**Construction of CART Models**

Admission CART models were constructed using a training dataset (N=288) and were assessed using a test dataset (N=515). A weighted sampling scheme was used to split the data, such that two-thirds of the dead/transplant outcome group (N=144) and the same number of the spontaneous survivors outcome group were randomly selected for inclusion within the training dataset. The remaining observations were contained within the test dataset. Sampling was conducted in this manner to increase the sensitivity of CART models while still maintaining high specificity and overall accuracy of the model. A CART model using the KCC variables was created, as well as a CART model with newly considered variables using the admission training data. Since trees can become too complex based on the specific training dataset used to build them, we pruned the CART models. A process in which unimportant variable splits are removed, pruning not only simplifies tree models, but also makes them more generalizable to external datasets. Admission trees were pruned by selecting the complexity parameter that minimized the cross-validated relative error rate.

Post-admission CART models were created using data from days 3-7 within the ALFSG registry. There was no readily available repeated measures software for CART at the time of analysis, so the post-admission data required reorganization prior to constructing CART models. Univariate analyses testing for a time dependency for each of the variables over days 3-7 were conducted using logistic regression accounting for repeated measures, and no trends were found (multiplicity-adjusted *p*-values > 0.05). Thus, a flat dataset with one observation per subject was compiled. For continuous predictors, the average of all non-missing values over days 3-7 was included in this dataset. For categorical indicator variables (coma grade I or II, MV, vasopressors use, RRT), if any of these were coded as “yes” for days 3-7, then the variable was coded as “yes” within the flat late-stage dataset. If all of these were coded as “no” for days 3-7 where data was collected, then the variable was coded as “no”, and otherwise the variable was coded as missing. Training data (N=146) and test data (N=354) were randomly split using a similar procedure as for admission data. Two-thirds of the dead/transplant outcome group (N=73) and the same number of the spontaneous survivors were randomly selected for inclusion within the training dataset, and the rest of the patients comprised the test dataset. After the post-admission CART models were developed, the trees were pruned using the complexity parameter that minimized the cross-validated relative error rate.

**Using CART Models for Prediction of Prognosis: An Example**

Suppose an APAP-ALF patient presents at hospital admission with the following characteristics: creatinine 3.2 mg/dL, INR 2.5, coma grade III, MELD 22, lactate 5.2 mmol/L, and the patient was on a ventilator. First, the admission KCC-CART prognosis model will be used. At the start, the creatinine of 3.2 mg/dL is greater than 1.5, so we proceed to Node 2. Next, INR of 2.5 is greater than 1.75, so we proceed to Node 4. Finally, coma grade is III, so we move to terminal Node 6, which estimates that the risk of death/transplant at 21 days is high (probability of death/transplant is 0.736). Using the admission NEW-CART, we move to Node 2, then to Node 4 and reach Node 6, where the probability of death/transplant is 0.761.

Suppose that the patient is still alive three days after hospital admission, and has the following characteristics: creatinine 1.4 mg/dL, INR 2.1, coma grade III, MELD 16, and lactate 3.7 mmol/L. For the post-admission KCC-CART model, we proceed to Node 2 then to Node 3, where the patient is identified as having a 0.353 probability of death/transplant by day 21. The post-admission NEW-CART model goes from Node 2 to 4 to 5, in which the patient is predicted as being in the death/transplant group at day 21 with probability 0.400.

**Limitations of CART Models**

Though CART models offered an alternative to current prognosis criteria, there are some limitations of this method. Firstly, CART models can create models, which are complex, and users must decide how to prune models, which may introduce bias. Using cross-validation, our CART models performed only slightly worse for the test dataset compared to the training dataset, which indicated that bias is likely minimal. Also, CART may have more variability compared to more complex statistical algorithms, such as ensemble models. This creates a more consistent prediction method, although the price for model stability is diminishing simplicity and interpretability. CART dominates ensemble methods in terms of simplicity, while still providing impressive accuracy and predictive performance. A final limitation of CART modeling was the lack of a repeated measures framework, which affected the late-stage models developed in this study. It would be preferable to use all of the data in its original form, rather than compressing multiple days of data per subject into one measure for each variable. Future development of repeated measures CART software may improve the predictive performance of CART models.
